# Elf1 Deficiency Impairs Macrophage Development in Zebrafish Model Organism

**DOI:** 10.3390/ijms26062537

**Published:** 2025-03-12

**Authors:** Qianli Tan, Jing Wang, Yimei Hao, Shizeng Yang, Biao Cao, Weijun Pan, Mengye Cao

**Affiliations:** 1 Shanghai Jiao Tong University School of Medicine, Shanghai 200025, China; tanqianli@sjtu.edu.cn (Q.T.); weijunpan@sibs.ac.cn (W.P.); 2 Shanghai Institute of Nutrition and Health, University of Chinese Academy of Sciences, Chinese Academy of Sciences, Shanghai 200031, China; wangjing2019@sinh.ac.cn (J.W.); ymhao@sinh.ac.cn (Y.H.); yangshizeng2018@sinh.ac.cn (S.Y.); caobiao@sinh.ac.cn (B.C.)

**Keywords:** ELF1, ETS, zebrafish, macrophage

## Abstract

The Ets (E-twenty-six) family of transcription factors plays a critical role in hematopoiesis and myeloid differentiation. However, the specific functions of many family members in these processes remain largely underexplored and poorly understood. Here, we identify Elf1 (E74-like factor 1), an Ets family member, as a critical regulator of macrophage development in the zebrafish model organism, with minimal impact on neutrophil differentiation. Through morpholino knockdown screening and CRISPR/Cas9-mediated gene editing, we demonstrate that Elf1 is critical for macrophage development and tissue injury responses. Specific overexpression of dominant-negative Elf1 (DN-Elf1) in macrophages demonstrated a cell-autonomous effect on macrophage infiltration. Furthermore, the overexpression of *cxcr4b*, a gene downstream of Elf1 regulation and essential for cell migration and injury response, significantly rescued this defect, indicating Elf1 as a key regulator of macrophage function. Our findings shed light on the roles of Elf1 in macrophage development and injury response and also highlight zebrafish as a powerful model for immunity research.

## 1. Introduction

Macrophages, essential components in the innate immune system, originate from hematopoietic progenitors derived from different hematopoietic origins [1]. Macrophages play crucial roles in immune responses, maintaining homeostasis, and facilitating tissue regeneration following injury. These functions include producing chemotactic factors to recruit inflammatory cells, clearing cellular debris, and secreting growth factors to promote wound healing, processes that rely heavily on macrophage infiltration at the injury site [2]. Despite the critical role of macrophages in immunity and tissue repair, the transcriptional mechanisms that govern their development and function remain poorly understood.

Multiple transcription factor families, including MAF (musculoaponeurotic fibrosarcoma) [3], IRF (interferon regulatory factor) [4], PPAR (peroxisome proliferator-activated receptor) [5], C/EBP (CCAAT/enhancer-binding protein) [6], and Ets (E-twenty-six) [7], regulate macrophage differentiation and maturation, as well as diverse functional responses, acting independently as well as through intricate interactions. For instance, IRF8 interacts with the transcription factor C/EBPα to influence the differentiation of myeloid progenitors into monocytes rather than neutrophils [8]. MAFB, a member of the MAF family, is critical for monocyte-to-macrophage differentiation and the polarization process [9]. Additionally, interactions between IRF8 and MAFB have been shown to regulate the cell cycle process and the differentiation of the human monocyte cell line THP-1 into macrophages [10].

The Ets family, characterized by a conserved DNA-binding ETS domain, is one of the largest and most widespread families of transcription factors across metazoans. To date, 28 Ets members have been identified in humans, 27 in mice, and 31 in zebrafish [11]. The Ets family plays a critical role in diverse cellular processes, including hematopoiesis, migration, and cell cycle regulation [12]. Based on a distance and parsimony analysis of the conserved ETS domains, the Ets family can be divided into 13 subgroups, including ETV (ETS variant), ELF (E74-like factor), and SPI (spleen focus forming virus proviral integration) [13]. Among these, the SPI subfamily member PU.1 is required for the development of multiple hematopoietic lineages and is involved in macrophage polarization and activation [14,15]. The ETV subfamily member ETV1 has been reported to regulate macrophage polarization in tumors [16]. These findings underscore the need for further research to explore the broader functions of other Ets family members, particularly in macrophages.

E74-like factor 1 (*ELF1*), a member of the Ets transcription factor family, was initially isolated from a human T-cell library using a probe targeting the ETS domain of *ETS1* [17]. Elf1 has since been shown to bind to the regulatory regions of several genes critical for immune cell development and activation, including GM-CSF (granulocyte–macrophage colony-stimulating factor) [18], *CD4* (cluster of differentiation 4) [19], and *c-Fes* (feline sarcoma oncogene) [20]. Elf1 is highly expressed throughout thymocyte development and in all stages of maturing thymocytes and T cells [21], with *Elf1* knockout mice exhibiting a significant reduction in NKT cells both in the thymus and peripheral tissues [22]. Research has also demonstrated that the overexpression of the ETS DNA-binding domain of ELF1 can selectively disrupt its normal function without affecting the activity of other ETS family members, such as ETS1 or ETS2 [23]. Despite its abundant expression in macrophage cell lines [21], the precise functional role of ELF1 in macrophages remains poorly characterized, highlighting a gap in understanding its potential regulatory functions in immune cell regulation.

The zebrafish genome exhibits significant homology with the human genome, making it an invaluable model for studying macrophage development and function. Similar to mammals, zebrafish hematopoiesis occurs in distinct waves from different anatomical sites, giving rise to various myeloid lineages, each regulated by specific genetic programs [24]. Previous studies have identified 31 ETS domain genes in the zebrafish genome through systematic analysis and demonstrated the functions of *erg*, *fli1*, and *etsrp* in hematopoiesis, vasculogenesis, and angiogenesis [25]. However, the role of Elf1 in zebrafish hematopoiesis remains unclear. Moreover, the optical transparency of zebrafish larvae enables high-resolution, real-time imaging of macrophage behaviors and interactions during the developmental process. Additionally, the advancement of gene editing technologies has further underscored the advantages of zebrafish as a model for genetic manipulation.

Given the complex transcriptional mechanisms governing macrophage regulation, it is likely that additional, yet unidentified, regulators are involved. To investigate this, we employed ATAC-seq and RNA-seq in zebrafish macrophages to systematically identify novel transcription factors that influence macrophage development. Our results demonstrate that Elf1 is a crucial regulator of macrophage development and their response to injury. By using morpholino-mediated knockdown and CRISPR/Cas9-mediated gene editing approaches, we investigate the effects of Elf1 deficiency on macrophage function in zebrafish larvae. Our findings reveal that Elf1 regulates macrophage activity through the transcriptional control of key downstream genes.

## 2. Results

### 2.1. Enrichment of Ets Family Binding Motifs in Zebrafish Macrophage-Accessible Chromatin

**Figure 1:**
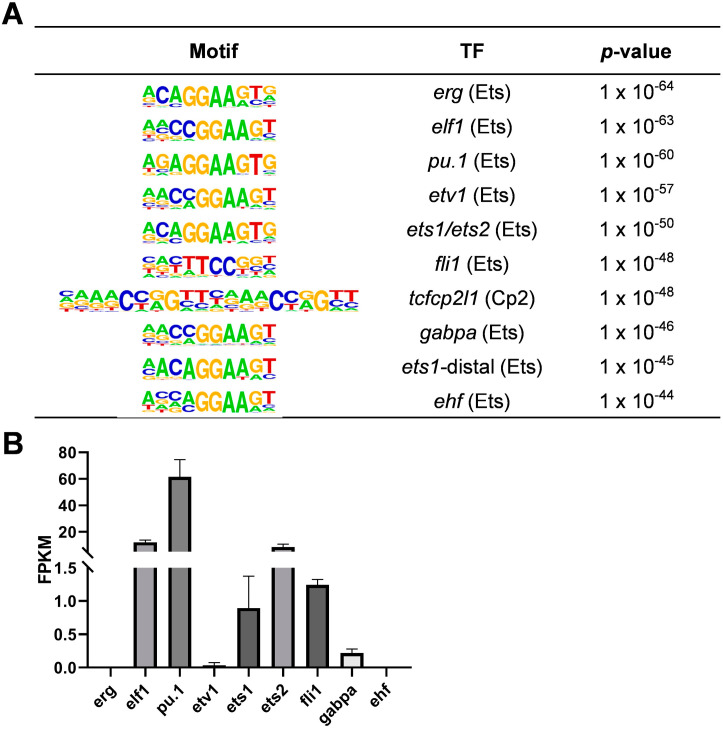
Transcription factor analysis of zebrafish macrophages via ATAC-seq and RNA-seq. (**A**) Motif enrichment analysis in zebrafish macrophages at 3 days post fertilization (dpf). The analysis reveals a strong enrichment of Ets family motifs, with 9 of the top 10 motifs belonging to the Ets family. (**B**) Expression levels of Ets family members in zebrafish macrophages, as determined via RNA-seq. Bar plots represent the mean ± standard error of the mean (S.E.M.).

To identify transcription factors critical for early macrophage development, we performed bulk ATAC sequencing once on zebrafish macrophages at 3 days post fertilization (dpf). After assessing data quality and analysis (See Section 4 and Figure S1A), we observed a significant enrichment of the GGAA motif, a known binding site for Ets family members, with 9 of the top 10 enriched motifs (by *p*-value) belonging to this family (Figure 1A). In the enriched results, we identified several Ets family transcription factors reported to control macrophage fate in zebrafish, including PU.1 [26] and Fli1 [27,28]. These findings not only support our results but also highlight the critical role of the Ets family in macrophage development and function. Given the diversity of Ets family members, we next assessed the expression of these factors in zebrafish macrophages through bulk RNA sequencing with three replicates (Figure 1B). After evaluating data quality and analysis (See Section 4 and Figure S1B,C), we found that *pu.1* and *fli1* were prominently expressed in macrophages, while *erg*, *etv1*, and *ehf* showed minimal expression. Notably, *ets1*, *ets2*, *gabpa*, and *elf1* exhibited significant expression (Figure 1B). However, their specific roles in macrophage biology remain poorly understood, warranting further investigation.

### 2.2. Selective Regulation of Macrophage Development by Elf1

**Figure 2:**
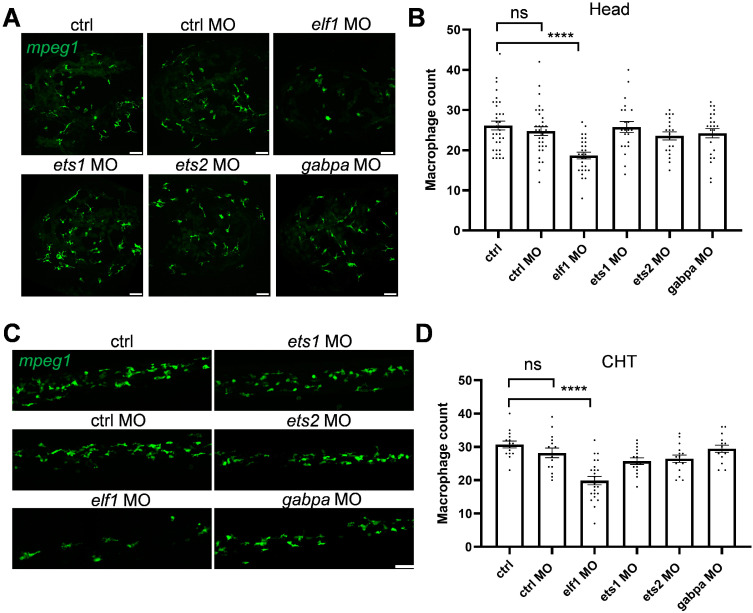
Morpholino knockdown-mediated screening of Ets family members in zebrafish macrophage development. (**A**) Representative confocal images showing macrophages in the head region of *Tg*(*mpeg1:eGFP*) zebrafish larvae at 3 dpf following morpholino-mediated knockdown of Ets family members. (**B**) Quantification of macrophage numbers in the head regions of the transgenic zebrafish shown in (**A**). Data were analyzed using one-way ANOVA followed by Dunnett’s test, with *n* ≥ 20, and shown as mean ± S.E.M. (**C**) Representative confocal images showing macrophages in caudal hematopoietic tissue (CHT) at 3 dpf following morpholino-mediated knockdown of Ets family members. (**D**) Quantification of macrophage numbers in the CHT of the transgenic zebrafish shown in (**C**). Data were analyzed using one-way ANOVA followed by Dunnett’s test, with *n* ≥ 15, and shown as mean ± S.E.M. Statistical significance is indicated as ns (*p* ≥ 0.05), **** *p* < 0.0001. Scale bar: 50 μm.

To investigate the regulatory roles of those Ets family members in macrophage development, we prioritized transcription factors based on their elevated expression profiles and motif enrichment patterns in zebrafish. Through morpholino (MO)-mediated knockdown, we aimed to uncover the specific functional contributions of these Ets factors to macrophage differentiation and activity during early development. Our results showed that only the Elf1 knockdown significantly reduced brain macrophage numbers, from an average of 26 to 18, while the knockdown of other Ets family members did not have a similar effect (Figure 2A,B). In addition, the Elf1 knockdown led to a decrease in macrophage numbers in the caudal hematopoietic tissue (CHT), whereas the knockdown of other Ets family members showed no impact (Figure 2C,D). These findings indicate that Elf1 specifically governs macrophage development during early zebrafish embryogenesis.

Additionally, the Elf1 knockdown did not affect somite length or overall embryonic development (Figure S2A,B), indicating that its role is specifically restricted to macrophage differentiation. Importantly, no significant alterations were observed in neutrophil numbers following Elf1 depletion (Figure S2C,D), underscoring Elf1′s selective involvement in macrophage development within the myeloid lineage, rather than affecting broader myeloid cell differentiation processes.

### 2.3. Macrophage Reduction in Elf1 Crispants

**Figure 3:**
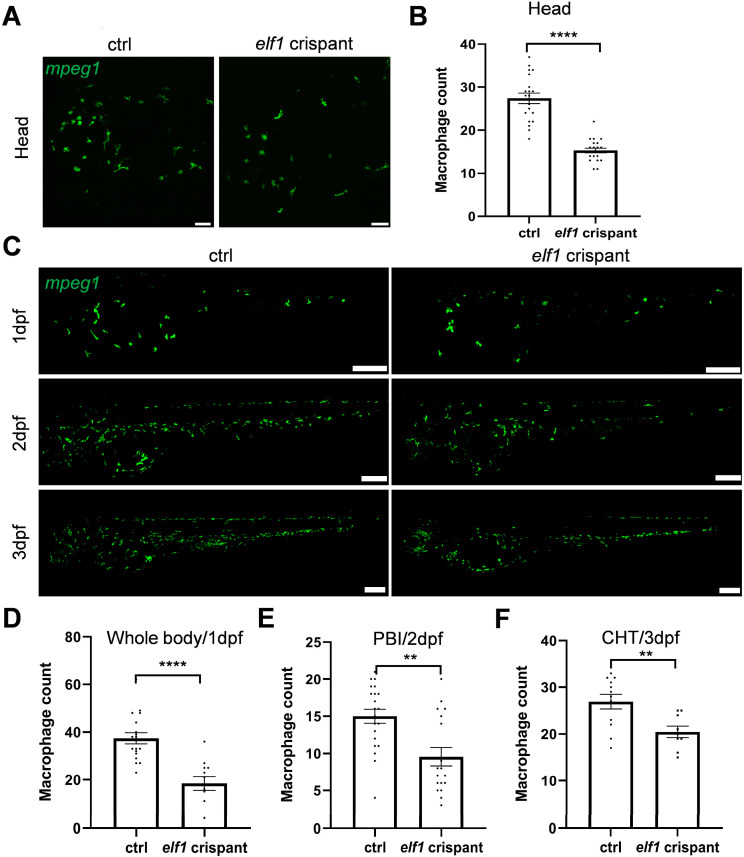
Reduction in macrophages in *elf1* crispants. (**A**) Representative confocal images showing macrophages in the head region in transgenic zebrafish at 3 dpf following CRISPR-mediated mosaic mutants. Scale bar: 50 μm. (**B**) Quantification of macrophage numbers within the brain regions of the transgenic zebrafish shown in (**A**). Unpaired, two-tailed Student’s *t*-test, with *n* ≥ 20, and results are shown as mean ± S.E.M. (**C**) Representative confocal images of macrophages in whole zebrafish larvae at 1, 2, and 3 dpf. Scale bar: 200 μm; imaging depth approximately 200 μm. (**D**–**F**) Quantification of macrophage numbers within specific regions of the transgenic zebrafish shown in (**C**), including the posterior blood island (PBI) and caudal hematopoietic tissue (CHT). Unpaired, two-tailed Student’s *t*-test for *n* ≥ 12, 18, and 9, respectively; results are shown as mean ± S.E.M. Statistical significance indicated as ** *p* < 0.01 and **** *p* < 0.0001.

To further elucidate the impact of Elf1 on macrophages, we employed multiple gRNAs to target *elf1*, successfully replicating the *elf1* knockout phenotype. Our results showed a significant reduction in macrophage numbers in both the brain and CHT regions of *elf1* crispants, similar to the phenotype observed in *elf1* morphants (Figure 3A–C,F). This further corroborates the essential role of Elf1 in macrophage development.

Given the multi-wave nature of zebrafish hematopoiesis during embryogenesis, we extended our analysis to macrophage development at 1 dpf and 2 dpf (Figure 3C). At 1 dpf, macrophage numbers were significantly reduced (Figure 3D), and at 2 dpf, a similar reduction was observed in the posterior blood island (PBI) region (Figure 3C,E). Collectively, these results highlight Elf1′s pivotal regulatory role in the development of macrophages throughout the transition from primitive to definitive hematopoiesis.

### 2.4. Impaired Macrophage Response to Injury in Elf1 Crispants

**Figure 4:**
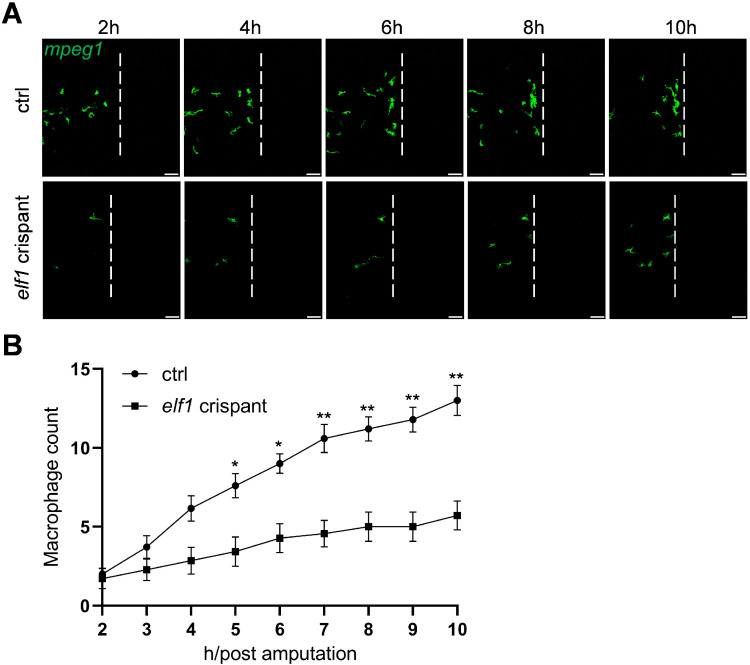
Macrophage behavior following tail amputation in *elf1* crispants. (**A**) Time-lapse images showing macrophage infiltration in controls and *elf1* crispants at 3 dpf. Imaging of the caudal fin region was initiated two hours post-amputation. The white dashed line marks the position of transection. Scale bar: 50 μm. (**B**) Quantification of macrophage numbers within the damage regions shown in (**A**). Data were analyzed using two-way ANOVA followed by Sidak’s tests, with *n* ≥ 7, and shown as mean ± S.E.M. Statistical significance indicated as * *p* < 0.05 and ** *p* < 0.01.

Macrophages play a crucial role in tissue repair and regeneration, playing a pivotal role in responding to injury signals. Transcription factors that regulate macrophage development also modulate their functional responses, such as activation during injury. Given Elf1′s critical role in macrophage development, we hypothesized that it might also impact macrophage activation in response to injury. To test this, we employed a tail amputation model in zebrafish larvae, which is a well-established assay for studying macrophage behavior during injury-induced inflammation [29]. Two hours post-amputation, we performed long-term imaging of the tail region to observe macrophage infiltration. The results showed that macrophage infiltration in *elf1* crispants was reduced compared to control zebrafish larvae throughout the observation period (Figure 4A,B). Notably, at 10 h post-amputation, macrophage numbers in the wounded area were reduced by 56% in *elf1* crispants compared to the control group, further highlighting the indispensable role of Elf1 in macrophage activation and response to tissue injury (Figure 4B). These results underscore Elf1’s crucial involvement not only in macrophage differentiation but also in regulating their functional responses during tissue repair.

### 2.5. Dominant-Negative Elf1 Reduces Macrophage Infiltration in a Cell-Autonomous Manner

**Figure 5:**
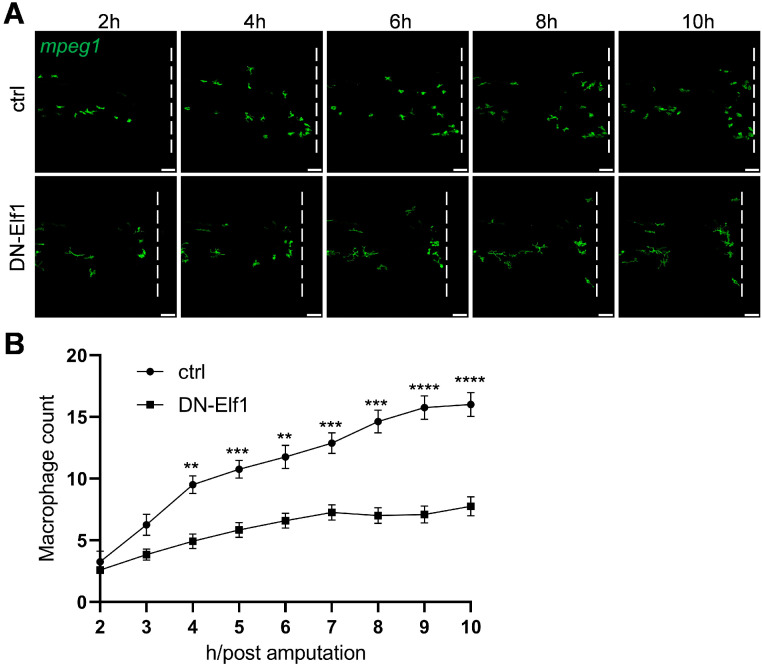
Overexpression of a dominant-negative form of Elf1 (DN-Elf1) in macrophages disrupts their response to injury. (**A**) Time-lapse imaging of macrophage infiltration at 3 dpf in control zebrafish and zebrafish expressing DN-Elf1 specifically in macrophages. The background transgenic line used was *Tg*(*mpeg1:Gal4FF/UAS:eGFPcaax*). Imaging of the caudal fin region was initiated two hours post-amputation. The white dashed line marks the position of transection. Scale bar: 50 μm. (**B**) Quantification of macrophage numbers within the damage shown in (**B**). Data were analyzed using two-way ANOVA followed by Sidak’s tests, with *n* ≥ 7, and shown as mean ± S.E.M. Statistical significance indicated as ** *p* < 0.01, *** *p* < 0.001, and **** *p* < 0.0001.

To test whether Elf1′s effects on macrophage function are cell-autonomous, we overexpressed a dominant-negative form of Elf1 (DN-Elf1) specifically in macrophages. Using Tol2-mediated transgenesis in *Tg*(*mpeg1:Gal4FF/UAS:eGFPcaax*) zebrafish larvae, we selectively disrupted Elf1 function in macrophages under the control of the macrophage-specific *mpeg1* promoter (Figure S3A,B) [30]. In the tail amputation assay, we observed a significant reduction in macrophage infiltration at the injury site in DN-Elf1-expressing zebrafish larvae. After 10 h post-amputation, fewer than half the number of macrophages were observed infiltrating the injury site compared to controls (Figure 5A,B). These results confirm that Elf1′s influence on macrophage activation is cell-autonomous.

### 2.6. Cxcr4b Mediates Elf1-Regulated Macrophage Infiltration

**Figure 6:**
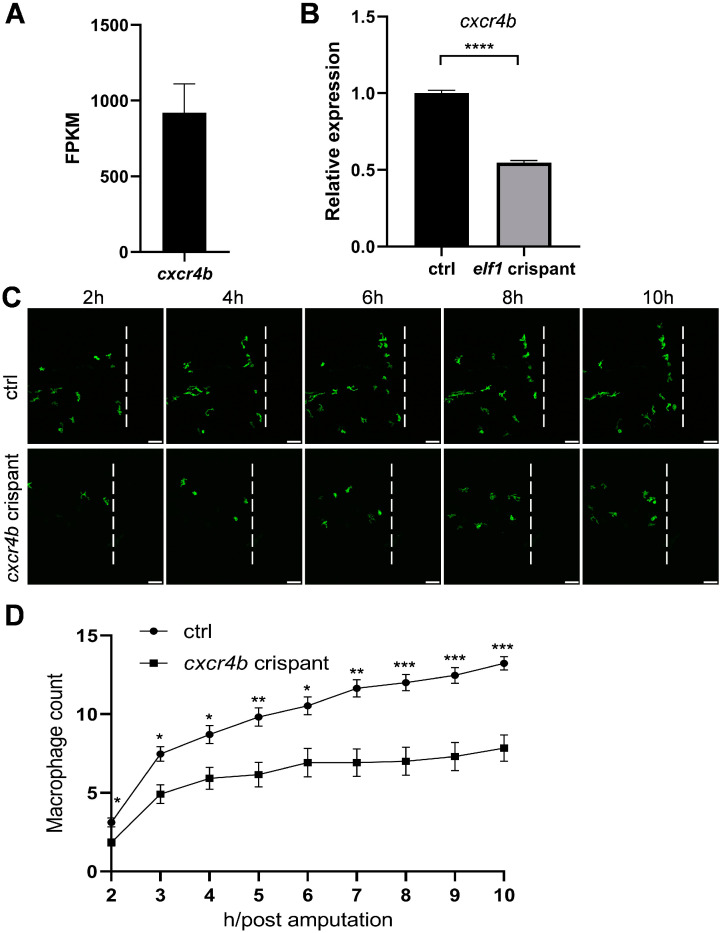
Macrophage behavior following tail amputation in *cxcr4b* crispants. (**A**) Expression levels of *cxcr4b* in zebrafish macrophages, as determined via RNA-seq. Bar plots represent the mean ± S.E.M. (**B**) Relative expression of *cxcr4b* in macrophages of *elf1* crispants compared to the control group at 3 dpf. Unpaired, two-tailed Student’s *t*-test results for *n* = 4 are shown as mean ± S.E.M. Statistical significance indicated as **** *p* < 0.0001. (**C**) Time-lapse images showing macrophage infiltration in controls and *cxcr4b* crispants at 3 dpf. Imaging of the caudal fin region was initiated two hours post-amputation. The white dashed line marks the position of transection. Scale bar: 50 μm. (**D**) Quantification of macrophage numbers within the damage regions shown in (**C**). Data were analyzed using two-way ANOVA followed by Sidak’s tests, with *n* ≥ 10, and shown as mean ± S.E.M. Statistical significance indicated as * *p* < 0.05, ** *p* < 0.01, and *** *p* < 0.001.

Given the cell-autonomous role of Elf1 in macrophage activation, we next explored the potential downstream targets of Elf1 that could mediate its effects on macrophage function. Notably, we found that *cxcr4b*, the zebrafish homolog of *cxcr4*, is as a key player in this process. Cxcr4b is highly expressed in macrophages (Figure 6A), and we identified an Elf1-binding motif within the open chromatin regions of the *cxcr4b* genetic locus. Cxcr4 is well established as a critical regulator of inflammatory responses, particularly in macrophage recruitment and tissue repair [31]. Based on these findings, we hypothesized that Elf1 might regulate macrophage function through Cxcr4b. Consistent with this, the qPCR analysis revealed a significant reduction in *cxcr4b* mRNA levels in macrophages from *elf1* crispants compared to controls, suggesting that *cxcr4b* is a downstream target of Elf1 (Figure 6B). To further test this hypothesis, we examined macrophage infiltration following tail amputation and observed a significant reduction in macrophage infiltration at the injury site following tail transection in *cxcr4b*-deficient zebrafish larvae. Approximately one-third fewer macrophages infiltrated the wound area compared to controls (Figure 6C,D). These results collectively support the hypothesis that Elf1 regulates the macrophage response to injury through Cxcr4b.

### 2.7. Rescue of Macrophage Infiltration via Cxcr4b Overexpression

**Figure 7:**
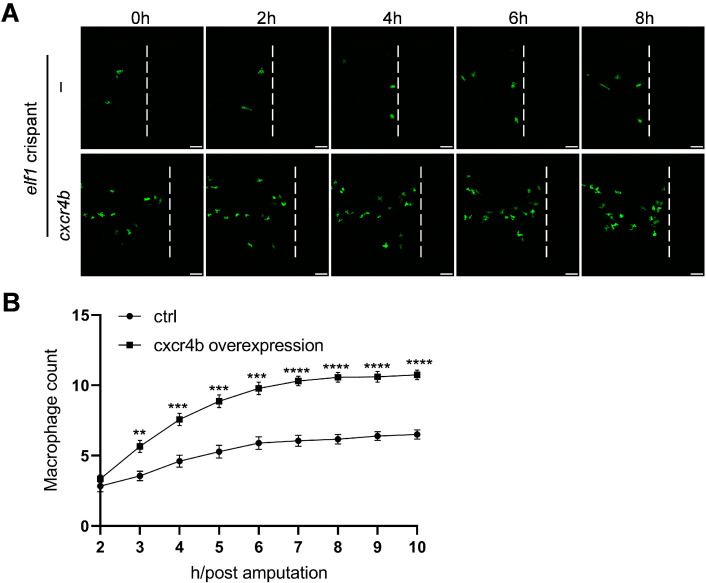
Ectopic expression of *cxcr4b* partially rescues macrophage function in *elf1* crispants. (**A**) Time-lapse images of macrophage infiltration in controls and *cxcr4b* ectopic expression in *elf1* crispant zebrafish larvae at 3 dpf. Imaging of the caudal fin region was initiated two hours post-amputation. The white dashed line marks the position of transection. Scale bar: 50 μm. (**B**) Quantification of macrophage numbers within the damage regions shown in (**A**). Data were analyzed using two-way ANOVA followed by Sidak’s tests, with *n* ≥ 18, and shown as mean ± S.E.M. Statistical significance indicated as ** *p* < 0.01, *** *p* < 0.001, and **** *p* < 0.0001.

To further validate the role of Cxcr4b in Elf1-mediated macrophage function, we conducted a rescue experiment. Given the significant reduction in macrophage infiltration in *elf1* crispants, we hypothesized that restoring Cxcr4b expression could rescue the impaired macrophage response. To test this, similar to the DN-Elf1 group, we specifically overexpressed Cxcr4b in macrophages in *elf1* crispants using the macrophage-specific *mpeg1* promoter (Figure S4A,B). Remarkably, compared to *elf1* crispants, the Cxcr4b overexpression group exhibited a notable increase in macrophage infiltration at the injury site (Figure 7A). Statistical analysis revealed a one-third increase in macrophage infiltration in the Cxcr4b overexpression group 10 h after tail amputation (Figure 7B). These findings confirm that Elf1 regulates macrophage response to injury by modulating Cxcr4b expression.

## 3. Discussion

In this study, we utilized ATAC-seq to reveal a notable enrichment of Ets family binding motifs within the chromatin-accessible regions of zebrafish macrophages, underscoring the critical role of Ets family transcription factors in macrophage development. Through morpholino knockdown screening, we first demonstrated that Elf1 selectively regulates macrophage development during the early zebrafish developmental process, without impacting neutrophil differentiation. Further validation with CRISPR/Cas9-mediated *elf1* mosaic mutants confirmed its essential role in both macrophage development and function. By overexpressing a dominant-negative form of Elf1, we demonstrated that Elf1′s influence on macrophage activation is cell-autonomous. Additionally, we found that Elf1 regulates macrophage responses to injury by modulating *cxcr4b* gene expression. Collectively, these findings highlight Elf1 as a critical transcription factor that specifically governs macrophage development and immune responses, underscoring its pivotal role in macrophage biology.

Both morpholino-based knockdown [32] and CRISPR/Cas9-mediated crispants [33] offer significant advantages for large-scale screening, time efficiency, cost-effectiveness, and simplicity. Despite each approach having inherent limitations, our core findings remained robust. By incorporating rigorous control comparisons and observing consistent results across both morpholino-based knockdown and CRISPR/Cas9-mediated crispant approaches, we further strengthened the reliability and interpretability of our findings, ensuring the validity of our results.

The Ets family transcription factors identified in our ATAC-seq enrichment analysis, including PU.1, Fli1, and Elf1, confirm the potential of the ATAC-seq approach in uncovering transcription factors involved in zebrafish macrophage development. In particular, Elf1, as a newly identified member, offers a novel research direction due to its association with macrophage development, further validating the effectiveness and reliability of ATAC-seq in studying zebrafish macrophage development.

The Ets family members are regulated by intricate networks of transcription factors and form functional complexes with specific partners to control downstream gene expression. Elucidating the regulatory mechanisms of ELF1 is essential for a deeper understanding of its pivotal role in gene regulation and cellular function. Previous studies have established a solid foundation for our findings. For example, PU.1 is essential for myeloid lineage development [34], and its high expression favors macrophage differentiation over neutrophil differentiation [35]. Consistent with this, the knockdown of PU.1 in the human monocyte cell line THP-1 results in a concurrent reduction in ELF1 expression [36], suggesting that ELF1 functions downstream of PU.1 in modulating macrophage development. Additionally, ELF1 has been shown to regulate the expression of key genes integral to macrophage biology. Notably, cooperative interactions between ELF1 and AP-1 play an important role in regulating inducible *GM-CSF* gene expression following human T-cell activation [18]. The human *FES* gene, which contains a strong myeloid-cell-specific promoter, is regulated by Sp1, PU.1, and ELF1 [19]. Similarly, the promoter and the macrophage-specific promoters of the mouse and human *CSF1R* genes are activated in RAW264 cells through the action of ETS2 and ELF1 [37]. These genes are essential for macrophage differentiation, activation, migration, survival, and proliferation, as well as immune responses [38,39,40]. Through its regulation of these genes, ELF1 deepens our insight into the molecular mechanisms through which its deficiency disrupts macrophage development and activation, revealing critical pathways involved in macrophage-specific functions and immune system dysregulation. ELF1 provides a clearer understanding of the molecular mechanisms underlying defects in macrophage development and activation. This evidence underscores ELF1 as a crucial regulator of macrophage-specific functions. However, a comprehensive exploration of the signaling networks and regulatory pathways is crucial to fully elucidate ELF1’s role in the regulation of myeloid lineage development.

During zebrafish embryogenesis, myeloid development is evolutionarily conserved across mammals. Zebrafish hematopoiesis consists of three distinct waves, each originating from different anatomical locations and governed by unique genetic programs [41,42]. The first wave, termed primitive hematopoiesis, generates myeloid cells and erythrocytes before 1 dpf [43]. Our results demonstrate a reduction in macrophages throughout the body at 1 dpf (Figure 3C,D), suggesting that Elf1 is involved in regulating primitive hematopoiesis. The second wave, intermediate hematopoiesis, occurs in the posterior blood island (PBI) from 24 to 48 hpf. This short wave produces committed erythromyeloid progenitors (EMPs), which differentiate into both erythroid and myeloid lineages [44]. Notably, we observed a reduction in macrophages within the PBI at 2 dpf (Figure 3C,E), indicating that Elf1 may also regulate intermediate hematopoiesis. Finally, definitive hematopoiesis is marked by the emergence of hematopoietic stem cells (HSCs), which then migrate to the caudal hematopoietic tissue (CHT) for proliferation and differentiation [45]. The reduction in macrophages in the CHT in both *elf1* morphants and crispants (Figure 2 and Figure 3) further underscores Elf1’s involvement in definitive hematopoiesis. Collectively, these findings suggest that Elf1 plays a pivotal role in regulating all three waves of hematopoiesis (Figure 3). However, further studies are essential to confirm these observations and unravel the detailed mechanisms underlying Elf1’s regulatory functions.

Macrophages are crucial for immune defense, tissue repair, and inflammation regulation [1]. Beyond its well-established role in macrophage development and injury response, ELF1 likely governs a broader spectrum of macrophage functions. For instance, ELF1 modulates *OAS1*, a gene crucial for antiviral responses following IFN stimulation [46], highlighting its potential role in immune regulation beyond macrophage activation. Additionally, Elf1 regulates *cxcr4b*, a gene that not only mediates macrophage and microglia responses to oncogene activation in the brain [47] but also plays an essential role in initiating the granuloma-associated proangiogenic program in zebrafish [48]. Given that Cxcr4b is a marker for M2 macrophages [49], ELF1 may influence macrophage polarization and tissue repair processes, broadening its regulatory role in macrophage biology. Further exploration of the regulation of macrophage function by ELF1 will be valuable for understanding the underlying mechanisms and exploring potential therapeutic applications.

Overall, our study identified a novel function of Elf1 in regulating macrophage development, mediated through its genetic downstream factor, *cxcr4b*. These findings provide new insights into Elf1’s broader role in myeloid lineage differentiation and its impact on immune system regulation.

## 4. Materials and Methods

### 4.1. Zebrafish Husbandry and Transgenic Lines

Zebrafish used in this study were raised using the ESEN system (Esen Technology Development Co., Ltd., Beijing, China) with the following conditions: circulating water temperature at 28.5–29 °C, pH between 6.5 and 7.5, and a light-dark cycle of 14 h of light followed by 10 h of darkness. The density was approximately 5 adult zebrafish per liter of water. Adult fish were fed twice daily with freshly hatched brine shrimp nauplii, in accordance with the guidelines of the Institutional Animal Care and Use Committee [50]. Transgenic lines used in this study include *Tg*(*mpeg1:eGFP*) for labeling macrophages and *Tg*(*mpx:GFP*) for labeling neutrophil, as described previously [30,51]. *Tg*(*mpeg1:Gal4FF/UAS:mCherrycaax*) and *Tg*(*mpeg1:Gal4FF/UAS:eGFPcaax*) lines were generated using the Tol2-mediated Gal4-UAS system [52,53]. Embryos were incubated at 28.5 °C in egg water [0.6% (*w/v*) baysalt and 0.01% (*w/v*) methylene blue], which was replenished daily. To prevent melanin pigmentation, embryos were incubated in egg water containing 0.045%1-phenyl-2-thiourea (PTU, Sigma-Aldrich, St. Louis, MO, USA) starting from 1 dpf, with daily water changes. For experiments using embryos prior to 48 hpf, the chorion was manually removed using fine forceps under a microscope (SZX10, Olympus, Tokyo, Japan). All animal care and experimental procedures were approved by the Animal Research Advisory Committee of the Shanghai Institute of Nutrition and Health, Chinese Academy of Sciences.

### 4.2. Sorting of Zebrafish Macrophages

Zebrafish macrophages were isolated for sequencing and quantitative PCR (qPCR) analysis. Selected embryos were washed with pre-chilled PBS, and the yolk sac tissue was removed by adding Ringer’s solution and gently pipetting the embryos. After centrifugation, embryos were dissociated with 0.25% trypsin (Sigma-Aldrich) at 30 °C. Once complete dissociation into single cells was confirmed under a microscope, digestion was terminated with 10% FBS in PBS (Sigma-Aldrich) and 2mM CaCl_2_ solution. The embryos were then washed with 1% BSA in PBS and prepared for sorting. Macrophages were isolated using the MoFlo Astrios EQ (Beckman Coulter, Brea, CA, USA). Control group embryo samples were prepared simultaneously in parallel for comparison.

### 4.3. ATAC and RNA Sequencing

For bulk ATAC sequencing, following cell sorting, sample quality was assessed using a cell counter (Thermo Fisher Scientific, Seattle, WA, USA). Only samples with ≥90% cell viability and ≤5% adhesion rate was subjected to subsequent operations. Nuclei were isolated from fresh samples, and nucleus morphology was examined under the microscope. Qualified nuclei were then translocated using the Nextera Kit (Illumina, San Diego, CA, USA). After purification, the samples were amplified, quality checked, and sequenced on the Illumina HiSeq platform (Illumina). The quality of raw reads was examined using FastQC (version 0.11.9). Clean reads were obtained by removing adaptor sequences before mapping. The clean reads were then aligned to reference genome sequences using the BWA program (version 0.7.17), with mapping statistics (Figure S1A). Unique mapped reads were used for Peak Calling via MASC2 (version 2.1.1), applying a q-value threshold of <0.05. Motif analysis was conducted with HOMER software (version 4.11) and matched with the existing Motif data in public databases. Due to the stringent requirements for nuclear quality and quantity in ATAC-seq, as well as the need for subsequent validation through a series of complementary assays, including RNA-seq and in vivo experiments, we performed a round of bulk ATAC-seq on zebrafish macrophages to identify transcription factors as the initial screening.

For bulk RNA sequencing, RNA samples were directly reversed amplified using SMARTer Ultra Low Input RNA for the Illumina Kit (Takara, Kyoto, Japan), followed by sequencing on the Illumina HiSeq 2500 platform (Illumina). The quality of raw sequencing reads was assessed using FastQC (version 0.11.9). Raw data were filtered using Seqtk (version 1.3) to remove adapter sequences, trim low-quality bases at 3′ ends, discard reads shorter than 25 bases, and eliminate ribosomal RNA reads. Genome comparison was performed with Hisat2 (version 2.0.4). And the number of reads was standardized for gene expression. The bulk RNA-seq experiment was performed in triplicate, and reproducibility among the three replicates was confirmed using Spearman’s correlation coefficient analysis (Figure S1B,C).

### 4.4. Morpholino Preparation and Microinjection

Morpholino antisense oligonucleotides (MOs) were designed and purchased from Gene Tools, LLC (Philomath, OR, USA). The *ets1* MO was previously reported in [54]. To design MOs, Ensemble accession numbers for target mRNA transcripts were identified based on differentially expressed transcripts and SNPs via PCR in zebrafish macrophages. The targets confirmed include *elf1* (ENSDART00000122369.4) and *ets2* (ENSDART00000161007.1), while the *gabpa* MO was designed to target all functional transcripts containing the ETS domain. Before ordering, as recommended by Gene Tools, LLC (Philomath, OR, USA), the specificity of the experimental MOs was verified in silico using the Ensemble BLAST/BLAT program (https://www.ensembl.org/, accessed on 25 January 2021). The MO sequences were input as nucleotide queries to identify any potential unintended targets in zebrafish cDNAs (transcripts/splice variants). Additionally, a standard control (Ctrl) MO was purchased as a negative control [55].

The lyophilized MOs were resuspended in nuclease-free water to make 3 mM stock solutions and aliquoted into 5 μL tubes. For experimental use, the following MOs were microinjected at the one-cell stage of zebrafish embryos: control MO (0.5 mM,1 nl), *elf1* MO (0.5 mM,1 nl), *ets1* MO (0.5 mM,1 nl), *ets2* MO (0.5 mM, 1 nl), and *gabpa* MO (0.25 mM, 1 nl). Higher concentrations caused deformities in zebrafish. The MO sequences are listed in Table S1.

### 4.5. Crispant Generation

The gene sequences of zebrafish *elf1* (ENSDART00000122369.4) and *cxcr4b* (ENSDARG00000041959) were obtained from Ensembl (www.ensembl.org, accessed on 20 November 2022). Targeted sequences were designed using CCTop-CRISPR/Cas9 online predictor (https://cctop.cos.uni-heidelberg.de:8043/, accessed on 21 November 2022) based on the composite score [56]. The gRNA sequences were input as nucleotide queries to identify any potential unintended targets in the zebrafish genomic sequence, and results were verified using Ensembl online tools. The modified gRNAs were ordered from Genescript, LLC (Nanjing, China). Compared with in vitro transcribed sgRNAs, the modified EasyEdit sgRNAs exhibited improved stability, reduced cytotoxicity, and higher editing efficiency [57].

Previous studies have demonstrated that utilizing multiple gRNAs in Cas9 ribonucleoprotein (RNP) complexes improves gene disruption efficiency and enables first-generation (F0) mosaic mutant zebrafish (crispants) to recapitulate the phenotypes of germline knockouts [58]. In this study, we employed multiple gRNAs to target genes and replicate the knockout phenotypes. The gRNAs and Cas9 protein (New England Biolabs, Ipswich, MA, USA) were co-microinjected into one-cell-stage embryos at a final concentration of 5 μM and a volume of 1 nl. To verify successful mutagenesis, genomic DNA was extracted from 10 randomly selected larvae per experimental group (*n* = 10/group), and PCR was performed using 2X PCR Master Mix (Beyotime, Shanghai, China). PCR products were sequenced to confirm the presence of the desired gene mutations. The gRNA sequences and genotyping details are listed in Table S1.

### 4.6. Plasmid Construction and Microinjection

The dominant-negative form of Elf1 (amino acids 200–297, NP_571234.2, NCBI) and the *cxcr4b* coding sequence (ENSDART00000061499.7, Ensembl) were amplified using PCR from cDNA derived from zebrafish larvae. For the pTol2: UAS-DN-Elf1-mCherry construct, the DN-Elf1 fragment was cloned into a modified Tol2-UAS-mCherry backbone. For the pTol2: UAS-cxcr4b-2A-mCherry construct, the *cxcr4b* coding sequences, followed by a 2A linker and mCherry in-frame, were cloned into a modified Tol2-UAS backbone. Restriction endonucleases and the KOD high-fidelity DNA polymerase were obtained from New England Biolabs. Vectors were ligated to linear segments using the Seamless Cloning Kit (Beyotime). Plasmids were purified using the phenol-chloroform method, and their concentration and purity were assessed using a Nanodrop spectrophotometer (Thermo Scientific). Embryos were injected with 20 pg of plasmid together with 15 pg of transposase mRNA. The transposase mRNA was synthesized in vitro using the SP6 mMessage mMachine Transcription Kit (Thermo Scientific). Primers used for plasmid construction and genotyping are listed in Table S1.

### 4.7. Total RNA Extraction and qPCR

Total RNA was extracted from sorted macrophages using the Trizol reagent and reverse-transcribed into cDNA using the HiScript IV 1st Strand cDNA Synthesis Kit (Vazyme, Nanjing, China). RNA concentrations were measured using a Nanodrop spectrophotometer (Thermo Scientific), and RNA quality was assessed through gel electrophoresis. Quantitative PCR was performed using SYBR Green Real-time PCR Master Mix (TOYOBO, Osaka, Japan) on a QuantStudio™6 Flex instrument (Thermo Scientific) with the comparative Ct (ΔΔCt) method. For qPCR, a 10 µL reaction mixture was prepared; it contained 15–25 ng of cDNA, 5 µL of SYBR Mix, and 0.3–0.4 µL of the forward and reverse primer mix (10 µM each), with the remainder filled with autoclaved Milli-Q^®^ water (Sigma-Aldrich). Gene expression levels were normalized to the endogenous control ef1a in zebrafish. The primers used for qPCR have been previously reported [36]. Data were analyzed using the ΔΔCt method. Primers used for qPCR analysis are listed in Table S1.

### 4.8. Imaging and Count

Confocal imaging of live transgenic zebrafish larvae was performed using the Zeiss LSM 880 inverted confocal microscope (Carl Zeiss, Oberkochen, Germany) or the Olympus IXplore inverted confocal microscope equipped with the Yokogawa CSU-W1 spinning disk unit (Olympus). Embryos were anesthetized with 0.03% Tricaine (Sigma-Aldrich) and mounted in 1% low-melt agarose for imaging. Image processing was carried out using Imaris 9.8.0 (Bitplane, Abingdon, Oxon, UK) software. For brain imaging, a depth of approximately 150 μm was captured, and macrophages in the eye region were excluded from the quantification. In the caudal hematopoietic tissue (CHT) region, macrophages were counted within a 500 μm area extending from the cloaca. In the tail region, macrophage numbers were quantified within 150 μm from the incision site.

### 4.9. Statistical Analysis

Statistical analysis was performed using GraphPad Prism 8 software (San Diego, CA, USA). A one-way analysis of variance (ANOVA) with Dunnett’s test was applied to assess morpholino screening results. The unpaired two-tailed Student’s *t*-test was used to assess the statistical significance of differences between two groups. The two-way ANOVA with Sidak’s tests was used for analysis of the tail amputation model results. Data are presented as mean ± standard error of the mean (S.E.M.). *p* values < 0.05 were considered statistically significant, with * *p* < 0.05, ** *p* < 0.01, *** *p* < 0.001, and **** *p* < 0.0001, and ns indicated no significance (*p* ≥ 0.05).

## Data Availability

The data that support the findings of this study are accessible upon reasonable request from the corresponding author.

## Supplementary Materials

The following supporting information can be downloaded at: https://www.mdpi.com/article/10.3390/ijms26062537/s1.
