# Supplementary material for: Elf1 Deficiency Impairs Macrophage Development in Zebrafish Model Organism
Source: Int J Mol Sci. 2025 Mar 12;26(6):2537. doi: 10.3390/ijms26062537 (PMC11942252; doi:10.3390/ijms26062537)
Supplement: Supplementary file 1 [file ijms-26-02537-s001.zip › ijms-3487693-supplementary.pdf]

A

ATAC-seq	Reads	Statistics	Mapping Ratio
	All reads	110970684	
Macrophage-ATAC	Mapped reads	109295081	98.5%
	Mapped unique reads	88848242	81.3%
	Mapped multi reads	20446839	18.7%

B

RNA-seq	Reads	Statistics	Mapping Ratio
Macrophage-1	All reads	41630316	
	Mapped reads	35157942	84.5%
	Mapped unique reads	34045917	96.8%
	Mapped multi reads	1112025	3.2%
Macrophage-2	All reads	42518102	
	Mapped reads	36145125	85.0%
	Mapped unique reads	35034737	96.9%
	Mapped multi reads	1110388	3.1%
Macrophage-3	All reads	43777366	
	Mapped reads	37650528	86.0%
	Mapped unique reads	36613701	97.2%
	Mapped multi reads	1036827	2.8%

C

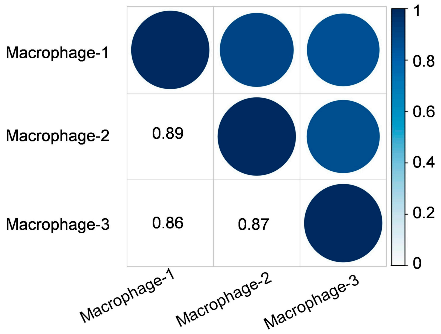

Figure S1. Quality assessment for ATAC-seq and RNA-seq. (A) Genome mapping statistics for ATAC-seq, showing that over 80% of reads were uniquely mapped, indicating high-quality data suitable for downstream analysis. (B) Genome mapping statistics for RNA-seq. (C) Spearman correlation analysis (colors representing correlation levels) demonstrating high similarity among triplicate samples.

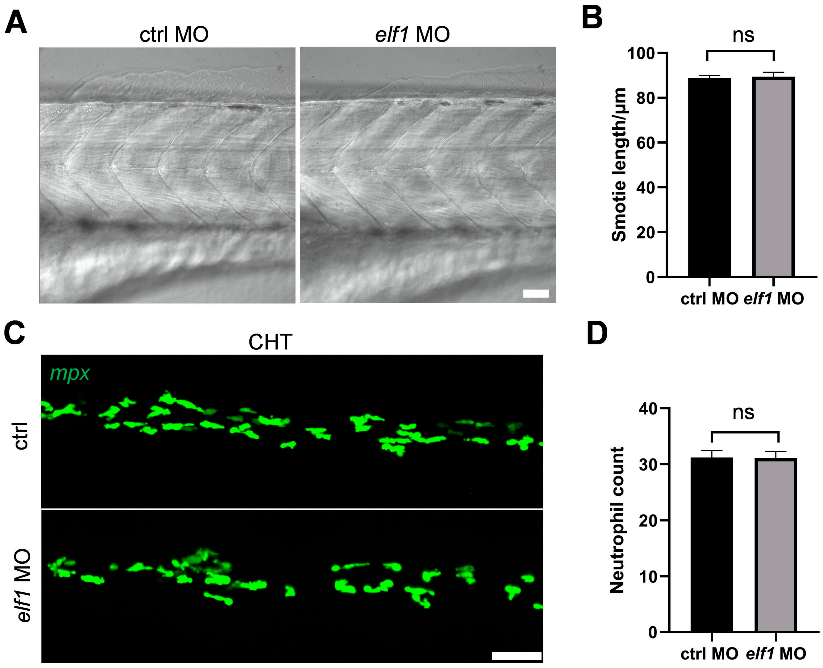

Figure S2. Effect of Elf1 knockdown on neutrophil development and zebrafish morphogenesis. (A) Representative confocal images of the trunk region in 3 dpf zebrafish larvae, captured by differential interference contrast (DIC) channel to assess developmental morphology. (B) Quantification of somite length in the trunk region of zebrafish larvae shown in (A). Unpaired, two-tailed Student's t-test, $n \geq 24$, mean ± S.E.M. Statistical significance is indicated as ns ($P \geq 0.05$). Scale bar: 50 μm. (C) Representative confocal images of neutrophil in CHT at 3 dpf. (D) Quantification of neutrophil numbers within the CHT, as shown in (C). Unpaired, two-tailed Student's t-test, $n \geq 25$, mean ± S.E.M.

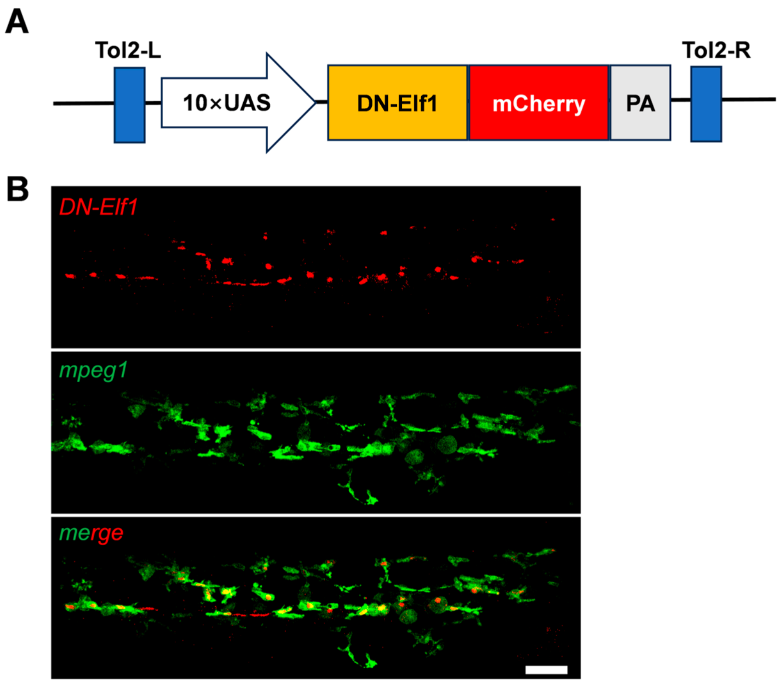

Figure S3. Expression of DN-Elf1 in macrophages. (A) Schematic of the DN-Elf1 overexpression plasmid construct. (B) Representative confocal images showing DN-Elf1 expression in macrophages at 3dpf. DN-Elf1 showed nuclear localization, as previously reported. Scale bar: 50 μm.

A

B

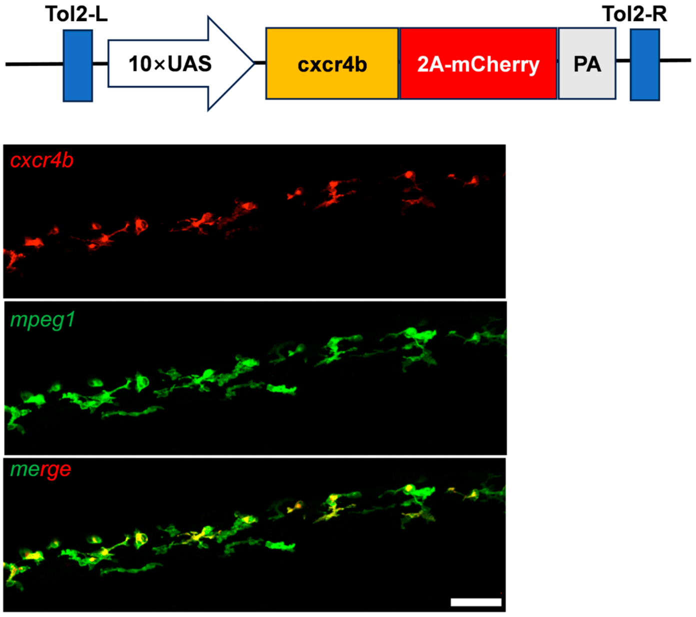

Figure S4. Expression of Cxcr4b in *elf1*-deficient macrophages. (A) Schematic of the *cxcr4b* overexpression plasmid construct. (B) Representative confocal images showing *cxcr4b* pattern expression in macrophages at 3dpf. Scale bar: 50 µm.

Table S1. MO, gRNAs and primers used in this study.

Name	Sequence (5'-3')
ctrl-MO	CCTCTTACCTCAGTTACAATTTATA
ets1-MO	GTCATGGTCACGCATTCAAACGTAC
ets2-MO	ATCCAGGTAAAAGCGACTCCATTTT
gabpa-MO	CTCCTCTGTCTCACTTTTCGACATC
efl1-MO	CTGACAGGCAGAGGGTTCCTACC
elf1-gRNA-1	GTACACCTGCATGAGGTCGG
elf1-gRNA-2	CGACCCTTCGGTGTTTCCAG
elf1-gRNA-3	AGAGAACATGATGGGAGACG
elf1-genotyping-F	TGCTGAAAGCTCCACATGACGG
elf1-genotyping-R	GAACAGCAGGTGATTCAAGTTTGC
cxcr4b-gRNA-1	CAGCTCTGACTCCGGTTCTG
cxcr4b-gRNA-2	GGGCGGTCGATGCAGTCAGC
cxcr4b-gRNA-3	CTACTGCAAGATAGCGGTCC
cxcr4b-gRNA-4	TGGTACCCATGCTCGAATTG
dnelf1-plasmid-F	TAACGGCCGCCAGTGTGCTGCCCGGGATGACCATCTACCTGTGGGAGTTCC
dnelf1-plasmid-R	AGGCTGAAGTTAGTAGCATCGATACCGTCGTCTTCATCAATAATGACCAG
cxcr4b-QPCR-F	GCGACCTCTCAGTCAGCAAT
cxcr4b-QPCR-R	TCACAAGCACCACAAGTCCA
ef1a-QPCR-F	TTCTGTTACCTGGCAAAGGG
ef1a-QPCR-R	TTCAGTTTGTCCAACACCCA
cxcr4b-plasmid-F	CGGCCGCCAGTGTGCTGCCCGGGATGGAATTTTACGATAGCATCATTTTAGACAAC
cxcr4b-plasmid-R	ACTCGTCAGTGCACTGGACGACTCTGATTCA
2A-mCherry-plasmid-F	CCAGTGCACTGACGAGTGGAAGCGGAGCTACTAACTTC
2A-mCherry-plasmid-R	ATGTATCTTATCATGTCGATATCTTACTTGTACAGCTCGTCCATG
